# Nucleic acid testing-optimized HIV diagnostic algorithms: a multimethod comparative effectiveness analysis in clinical practice

**DOI:** 10.3389/bjbs.2026.15887

**Published:** 2026-07-29

**Authors:** Defu Yuan, Fei Zhao, Li Li, Xiaoxue Tian, Xizhao An, Zhenglai Ma, Ying Gao, Michelle Muxiao Wang, Lijun Sun, Hao Wu, Tong Zhang, Bin Su, Bei Wang, Lifeng Liu

**Affiliations:** 1 Department of Epidemiology and Health Statistics, Key Laboratory of Environmental Medicine Engineering of Ministry of Education, School of Public Health, Southeast University, Nanjing, China; 2 Beijing Key Laboratory for HIV/AIDS Research, Sino-French Joint Laboratory for HIV/AIDS Research, Clinical and Research Center for Infectious Diseases, Beijing Youan Hospital, Capital Medical University, Beijing, China; 3 Haidian Foreign Language Academy, Beijing, China; 4 Center for Infectious Diseases, Beijing Youan Hospital, Capital Medical University, Beijing, China; 5 Central Laboratory, Beijing Youan Hospital, Capital Medical University, Beijing, China

**Keywords:** diagnosis, DNA, ELISA, HIV, RNA

## Abstract

**Background:**

Global and Chinese efforts still face significant gaps in achieving the first 95% of the “95-95-95” target, with persistently high and rising rates of late HIV diagnosis. This study evaluates RNA/DNA quantification, RNA qualitative, and ELISA assays to optimize HIV testing strategies.

**Methods:**

A prospective cross-sectional study evaluated 215 first-time HIV testers from June 2024 to May 2025. Using clinical diagnosis as the reference standard, we assessed four methods' sensitivity, specificity, and subgroup performance, with tandem testing strategies simulation for optimal detection.

**Results:**

DNA quantitative detection demonstrated optimal performance with 100% sensitivity and specificity. RNA quantitative assay showed 99.02% sensitivity and 100% specificity, while ELISA achieved 99.02% sensitivity and 98.23% specificity. RNA qualitative testing exhibited 99.02% sensitivity but lower specificity (75.22%). ROC revealed superior diagnostic performance for DNA quantitative (AUC = 1.000) and RNA quantitative (AUC = 0.995) compared to RNA qualitative (AUC = 0.871). All methods maintained consistent sensitivity across CD4^+^ T cell levels. Simulation of tandem strategies identified ELISA combined with DNA quantitative testing as optimal (net sensitivity: 99.02%, net specificity: 100%, total tests: 318). For 18 WB-indeterminate samples, DNA/RNA quantitative methods achieved 100% diagnostic accuracy, outperforming RNA qualitative (94.44%) and ELISA (83.33%).

**Conclusion:**

DNA quantitative detection shows high diagnostic value in initial HIV testing, overcoming challenges from undisclosed ART-induced RNA suppression and resolving WB-indeterminate misclassifications to reduce late diagnosis risks. This study supports Nucleic acid tests into diagnostic algorithms and validates the superior performance of ELISA screening followed by DNA confirmation, offering actionable strategies to shorten diagnostic delays and advance national AIDS control objectives.

## Introduction

According to the latest UNAIDS report, the global number of newly reported HIV infections remained stable in 2024, with 1.3 million new cases reported annually, bringing the total population of people living with HIV (PLWH) to 40.8 million [1]. China reported 100,000 new HIV cases in the same year, with the total number of PLWH reaching 1.35 million [2]. The global and Chinese achievements of the “95-95-95” targets stand at 87%-89%-94% [1] and 84%-93%-97% [3], respectively. Although both global and national figures for treatment rates and viral suppression have approached or even exceeded the 2030 targets, the first 95% remains the current shortfall, with estimates indicating approximately 5.3 million and 200,000 PLWH remain undiagnosed globally and in China, respectively. The National AIDS Prevention and Control Plan (2024-2030) emphasizes improving HIV diagnosis and treatment outcomes, requiring the proportion of diagnosed cases aware of their infection status to reach over 90% by 2025 and exceed 95% by 2030 [4], making case identification a critical focus for future HIV control efforts.

Previous studies defining late presentation as initial CD4^+^ T cell count <350 cells/µL or presence of AIDS-defining events at diagnosis reveal that approximately 50% of newly diagnosed PLWH globally are late presenters [5]. China reported a 60.56% late-presentation rate from 2013 to 2023, showing an increasing annual trend [6]. Late diagnosis not only elevates HIV transmission risks but also significantly increases AIDS-related morbidity and mortality [7, 8]. This situation severely compromises patients' quality of life and life expectancy while imposing substantial burdens on global public health systems and economies [9]. The persistent challenge of late diagnosis underscores the urgent need to develop and implement improved diagnostic protocols for earlier HIV detection.

The current HIV diagnostic criteria in China require reactive HIV antibody screening tests plus any one of the following: (1) positive HIV antibody confirmation test; (2) positive HIV nucleic acid qualitative test; (3) HIV RNA >1000 copies/mL, or two consecutive positive nucleic acid tests (NAT) with epidemiological history/AIDS-related clinical manifestations, or positive HIV isolation test [10, 11]. The most common confirmation method involves positive screening followed by Western blot (WB) confirmation. However, WB demonstrates a median window period exceeding 40 days, significantly longer than the 11.5-day median window period of nucleic acid testing [12]. Nationwide reports indicate nearly 10% of reactive screening samples show indeterminate WB results [13, 14], with most corresponding individuals ultimately diagnosed as HIV-positive through supplemental testing [15, 16], highlighting limitations of traditional confirmation protocols and underscoring the need for more accurate diagnostic algorithms.

In 2023, the U.S. CDC updated its HIV testing algorithm to: (1) HIV-1/2 antigen/antibody combination assay; (2) HIV-1 nucleic acid test; (3) HIV-1/2 antibody differentiation assay [17]. Studies suggest this modified approach reduces total test numbers and turnaround time while maintaining diagnostic accuracy [18], affirming nucleic acid testing’s crucial role in timely diagnosis. Analyses of individuals with indeterminate/negative WB results revealed that >99% of those with viral load exceeding detection limits eventually received positive diagnoses [15, 16], further supporting nucleic acid testing’s potential in HIV confirmation. However, China still lacks direct evidence for implementing nucleic acid testing in confirmation protocols. While HIV reservoir size correlates with clinical outcomes [19], and the first globally approved DNA quantification kit (launched September 2024) enables standardized HIV-DNA testing for reservoir assessment, its performance in initial diagnosis remains unstudied.

Building upon existing diagnostic frameworks, this study aims to evaluate enzyme-linked immunosorbent assay (ELISA), RNA quantification, DNA quantification, and RNA qualitative testing for identifying HIV infection among treatment-naïve patients in China. Our findings could inform optimized testing strategies to facilitate early diagnosis, support China’s HIV/AIDS containment initiatives, and contribute to achieving the 2030 global target for ending AIDS.

## Materials and methods

### Study design

This prospective cross-sectional study aimed to evaluate the performance of ELISA, HIV RNA quantitative assay, HIV RNA qualitative assay, and HIV DNA quantitative assay in identifying HIV infection among first-visit testers. All enrolled participants underwent standardized HIV testing and diagnostic procedures [10, 11]. Infection status was determined through a comprehensive evaluation integrating laboratory results, clinical manifestations, and epidemiological exposure history, serving as the gold standard in this study. All participants classified as negative underwent at least 3 months of follow-up to exclude potential acute-phase interference with conventional diagnostic protocols.

### Participants and inclusion criteria

The study population comprised individuals undergoing their first HIV testing at Beijing Youan Hospital between June 2024 and May 2025. Inclusion criteria were: (1) age ≥18 years at testing; (2) self-reported no prior antiretroviral therapy (including pre-/post-exposure prophylaxis); and (3) voluntary participation with informed consent.

### Sample size estimation

Based on previous studies reporting ELISA sensitivity (99%) and specificity (98%), and DNA/RNA assays’ sensitivity (100%) and specificity (99%) [20], we calculated minimum sample sizes using MedCalc software (version: 23.2.1) with α = 0.05 and 5% margin of error. Required sample sizes were 95 (43 positive, 52 negative) for ELISA and 79 (36 positive, 43 negative) for DNA/RNA assays. To ensure sufficient power for all comparisons, the larger ELISA-derived sample size (n = 95) was adopted. Considering Bonferroni correction for multiple comparisons (adjusted α = 0.05/6 = 0.0083), the minimum sample size was 170 (77 positive, 93 negative).

### Sample collection and processing

Peripheral venous blood samples (10 mL) were collected from eligible participants using standardized EDTA-K2 (dipotassium ethylenediaminetetraacetic acid) anticoagulant tubes. Blood processing commenced immediately after collection. Anticoagulated blood was centrifuged at 3,000 rpm for 15 min, followed by stratified separation. Three aliquots were prepared: two 1.5 mL plasma aliquots (upper layer), one 0.5 mL lymphocyte-enriched fraction (middle layer), and residual components. All aliquots were labeled with identical codes and stored at −80 °C.

### Ethics approval

This study was approved by the Ethics Committee of Beijing Youan Hospital Affiliated to Capital Medical University (No. 2024-145). Clinical data and sample collection procedures complied with institutional ethical standards, all individuals provided informed consent to participate in this study. The raw data did not contain any personal identifying information that could be linked to a particular individual and was anonymized before use.

### Laboratory methods

Plasma HIV-1 viral load quantification was performed using the Abbott m2000sp™ automated system with Abbott RealTime HIV-1 reagents (National Medical Device Registration No. 20180021). RNA was extracted via automated magnetic bead-based methods, followed by reverse transcription real-time fluorescent PCR (RT-PCR) with TaqMan probes on the m2000rt platform, achieving a quantification range of 40–10^7^ copies/mL. Internal controls monitored amplification efficiency throughout the process.

Plasma HIV-1 RNA qualitative detection utilized a multiplex real-time PCR kit (Wantai Biological Pharmacy, Beijing). Automated nucleic acid extraction was performed using magnetic beads, followed by simultaneous HBV/HCV/HIV detection on the ABI 7500 platform via multiplex TaqMan probes (HIV target: Cy5 channel). Competitive internal controls (VIC/HEX channel) monitored extraction/amplification efficiency, with UNG enzyme contamination prevention and a sensitivity of 40 IU/mL. Results were interpreted as positive when Ct values ≤37.

Lymphocyte-enriched HIV-1 DNA quantification employed a real-time PCR kit (Hightest Biotech, Guangzhou). Genomic DNA was extracted using Tianlong magnetic bead-based kits (Model T324) on the GeneRotex96 system. qPCR detection on the ABI 7500 platform utilized dual probes (FAM channel targeting HIV-1 conserved sequences; VIC channel quantifying nucleated cells) with dUTP/UDG contamination control. A standard curve was established, and results were normalized to HIV-1 DNA copies per 10^6^ nucleated cells. Validity required negative control non-amplification, acceptable positive control values, and standard curve correlation coefficients |r| ≥0.98.

Plasma HIV-1 antibody and P24 antigen detection used a commercial ELISA kit (Wantai Biological Pharmacy, Beijing) based on dual-antigen sandwich (antibody) and dual-antibody sandwich (P24) principles. After sample equilibration, reactions were performed in pre-coated microwells at 37 °C, followed by washing, enzyme conjugation, TMB substrate incubation, and absorbance measurement at 450 nm. Cut-off values were defined as the negative control mean + 0.12.

### Data collection

Demographic characteristics, baseline CD4^+^ T cell counts, and historical WB results were retrospectively retrieved from the hospital’s Electronic Medical Record (EMR) system. Details regarding the data extraction, missing data, and specific WB diagnostic thresholds are provided in Supplementary Material 1.

### Statistical analysis

Data was analyzed using R (version: 4.4.2). Normally distributed variables were expressed as mean ± SD (x̄ ± s) and compared via t-tests; non-normal variables were described as median (IQR) [M (P25, P75)] and analyzed using rank-sum tests. Categorical variables were compared via χ2 or Fisher’s exact tests. Diagnostic performance (sensitivity, specificity, accuracy, Youden index) was evaluated, with Cochran’s Q and McNemar tests comparing sensitivities across methods. ROC curve analysis with DeLong’s test assessed AUC differences. Diagnostic agreement was quantified via Kappa coefficients (0.81–1.00: near-perfect agreement). Stratified McNemar tests evaluated CD4^+^ T cell subgroup sensitivity differences. Net sensitivity/specificity and testing frequency for serial algorithms were calculated. Bonferroni correction (α = 0.0083) adjusted for six pairwise comparisons; all tests were two-tailed, with P ≤ 0.05 considered statistically significant unless specified.

## Results

### Study population

This study enrolled 215 participants (Table 1), including 113 HIV-negative (52.56%) and 102 HIV-positive individuals (47.44%). The HIV-positive group exhibited a significantly younger median age compared to the HIV-negative group (36.00 vs. 46.00 years, Z = −4.905, P < 0.001), with no significant sex distribution difference (male proportion: 98.04% [100/102] vs. 95.58% [108/113], Fisher’s exact test P = 0.446). Among HIV-positive participants, transmission routes were predominantly homosexual transmission (74.51%, 76/102), followed by heterosexual transmission (8.82%, 9/102) and other routes (16.67%, 17/102). Viral load analysis revealed 75.49% (77/102) had ≥5,000 copies/mL, 9.80% (10/102) 1,000–4,999 copies/mL, and 13.73% (14/102) detectable levels <1,000 copies/mL. Immunological status showed 41.18% (42/102) with CD4^+^ T cells >350 cells/μL, 30.39% (31/102) 200–350 cells/μL, and 18.63% (19/102) <200 cells/μL. CD4/CD8 ratios were distributed as 49.02% (50/102) ≥0.4, 25.49% (26/102) 0.2 to <0.4, and 15.69% (16/102) <0.2.

**TABLE 1:** Characteristics of 215 individuals who accept the HIV diagnosis testing.

Variables	HIV status		Z/χ^2^	P value
	Negative	Positive		
Age	46.0 (36.5–55.0)	36.0 (28.0–46.3)	−4.905	<0.001
Gender			a	0.446
Male	108	100	​	​
Female	5	2	​	​
Infection route				
Heterosexual	-	9	​	​
Homosexual	-	76	​	​
Others	-	17	​	​
Viral load (copies/mL)				
≥5000	0	77	​	​
1000-4,999	0	10	​	​
Detected-999	0	14	​	​
TND	113	1	​	​
CD4^+^ T cell (cells/µL)				
>350	-	42	​	​
200-350	-	31	​	​
<200	-	19	​	​
Missing	-	10	​	​
CD4/CD8				
≥0.4	-	50	​	​
0.2 to <0.4	-	26	​	​
<0.2	-	16	​	​
Missing	-	10	​	​
Total	113	102	​	​

a Fisher’s Exact Test (2 cells have expected count less than 5).

### Diagnostic performance

Concurrent testing of 215 samples using RNA quantification, RNA qualitative, DNA quantification, and ELISA methods revealed distinct diagnostic accuracies (Table 2). DNA quantification demonstrated optimal performance with 100% sensitivity (102/102), 100% specificity (113/113), 100% accuracy, and a Youden index of 1. RNA quantification showed near-equivalent efficacy (sensitivity 99.02% [101/102], specificity 100% [113/113], accuracy 99.53%, Youden index 0.99). ELISA achieved 99.02% sensitivity (101/102), 98.23% specificity (111/113), 98.60% accuracy, and a Youden index of 0.97. RNA qualitative testing exhibited lower specificity (75.22% [85/113]) despite 99.02% sensitivity (101/102), yielding 86.51% accuracy and a Youden index of 0.74.

**TABLE 2:** Diagnostic performance of four assays for HIV detection.

Methods	Sensitivity (%)	Specificity (%)	Accuracy (%)	Youden index
RNA quantification	99.02	100.00	99.53	0.99
RNA detection	99.02	75.22	86.51	0.74
DNA quantification	100.00	100.00	100.00	1.00
ELISA	99.02	98.23	98.60	0.97

Comprehensive statistical testing confirmed the robust non-inferiority among the evaluated methods (all P > 0.05, detailed in Supplementary Material 1, Supplementary Table S1), suggesting clinical equivalence in detecting HIV infections, with DNA quantification showing zero missed diagnoses.

**FIGURE 1:**
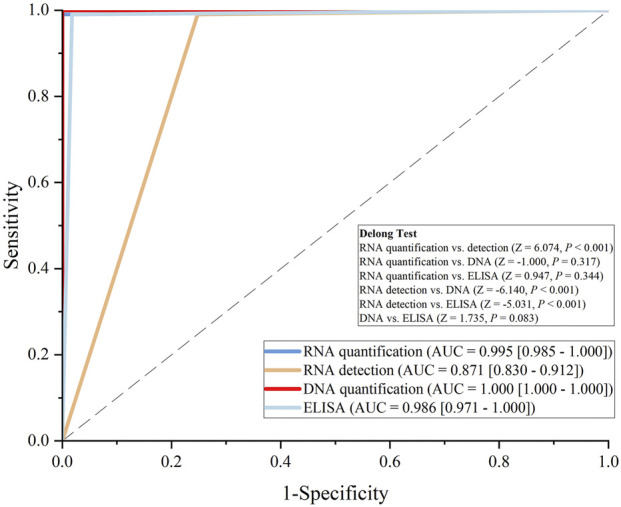
Comparative ROC curve analysis of HIV RNA quantification, qualitative RNA detection, DNA quantification, and ELISA for initial HIV diagnosis.

ROC curve analysis (Figure 1; Supplementary Table S2) highlighted excellent diagnostic efficacy for all methods. AUC values were 0.995 (95% CI: 0.985–1.000) for RNA quantification, 0.871 (95% CI: 0.830–0.912) for RNA qualitative, 1.000 (95% CI: 1.000–1.000) for DNA quantification, and 0.986 (95% CI: 0.971–1.000) for ELISA, all significantly differing from 0.5 (P < 0.001). Unadjusted Delong tests showed DNA quantification outperformed RNA qualitative (AUC difference: 12.9%, P < 0.001), RNA quantification surpassed RNA qualitative (12.4%, P < 0.001), and ELISA exceeded RNA qualitative (11.5%, P < 0.001). Post-correction, DNA vs. RNA qualitative, RNA quantification vs. RNA qualitative, and ELISA vs. RNA qualitative retained statistical significance (P < 0.001 < 0.0083).

### False-negative and false-positive outcomes

Among 102 HIV-positive samples, RNA quantification and RNA qualitative testing each produced one false-negative result (same patient, CD4^+^ T cell counts>700 cells/μL). ELISA yielded one false-negative case with a viral load of 1,188,617 copies/mL (exceeding the group mean of 195,566 copies/mL) and CD4^+^ T cell counts 155 cells/μL. DNA quantification showed no false negatives. For 113 HIV-negative samples, RNA and DNA quantification produced zero false positives, ELISA generated two (WB-indeterminate, later confirmed negative), and RNA qualitative testing yielded 28 false positives without overlapping with ELISA cases.

### Inter-assay agreement

**FIGURE 2:**
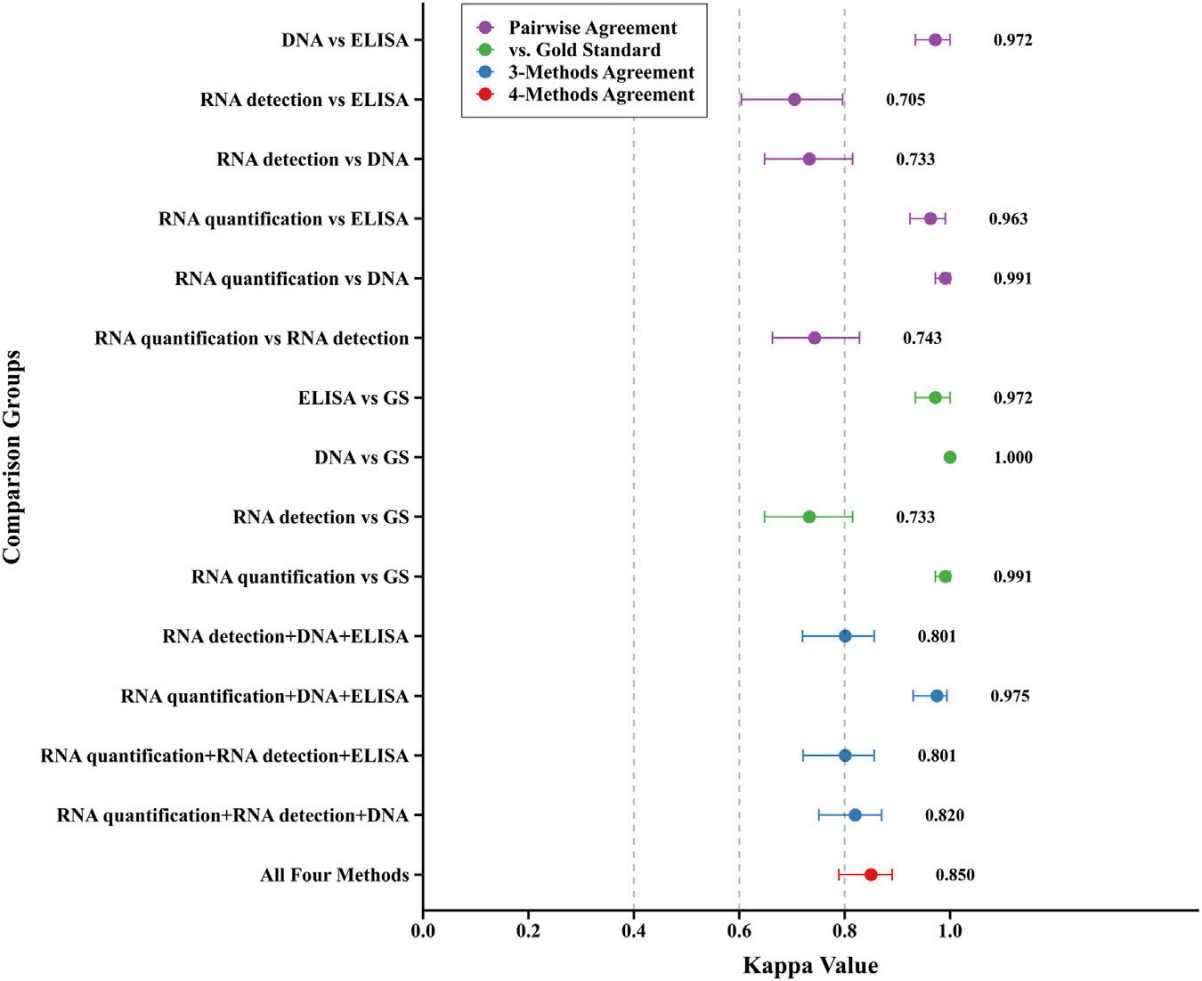
Kappa analysis of agreement between HIV diagnostic methods and reference standard.

Kappa analysis (Figure 2; Supplementary Table S3) revealed near-perfect overall agreement among all four assays (Kappa = 0.850). DNA quantification showed perfect concordance with the gold standard (Kappa = 1.000), followed by RNA quantification (Kappa = 0.991) and ELISA (Kappa = 0.972). RNA qualitative testing demonstrated substantial agreement (Kappa = 0.733). Pairwise comparisons indicated near-perfect agreement between RNA quantification and DNA (Kappa = 0.910), RNA quantification and ELISA (Kappa = 0.963), and DNA and ELISA (Kappa = 0.972). RNA qualitative testing showed substantial agreement with other methods (Kappa = 0.705–0.743).

### Stratified analysis

Subgroup analysis of 102 HIV-positive patients by CD4^+^ T cell counts (Supplementary Tables S4, S5) demonstrated consistent sensitivity across assays: CD4^+^ T cell counts >350 cells/μL (42 cases): RNA quantification (41/42), RNA qualitative (41/42), DNA (42/42), ELISA (42/42); CD4^+^ T cell counts 200–350 cells/μL (31 cases): all methods detected 31/31; CD4^+^ T cell counts <200 cells/μL (19 cases): RNA/DNA detected 19/19, ELISA 18/19; missing CD4^+^ T cell counts data (10 cases): all methods detected 10/10.

### Multi-assay algorithm

Simulated multi-assay strategies (Supplementary Table S6) achieved high specificity (≥99.56%), with several combinations reaching 100%. Net sensitivity ranged from 98.05% to 99.02%. The ELISA-DNA tandem strategy achieved optimal performance (sensitivity 99.02%, specificity 100%, 318 total tests). Nucleic acid-based initial screening (RNA/DNA) combined with secondary testing also achieved 99.02% sensitivity and 100% specificity, requiring 316–344 tests. RNA-DNA and RNA-RNA qualitative tandems required the fewest tests (316), while ELISA-RNA/DNA combinations required 344.

### WB-indeterminate cases

Among 18 WB-indeterminate samples (10 negative, eight positive), DNA and RNA quantification demonstrated perfect diagnostic accuracy (100% sensitivity, specificity, accuracy, Youden index 1.00). RNA qualitative testing showed 100% sensitivity, 90% specificity, 94.44% accuracy, and a Youden index of 0.90. ELISA exhibited 87.50% sensitivity, 80% specificity, 83.33% accuracy, and a Youden index of 0.68.

## Discussion

This study systematically evaluated the performance of four detection methods (RNA quantification, RNA qualitative, DNA quantification, and ELISA) in identifying HIV infection among first-time testers. The results demonstrated excellent diagnostic efficacy across all four methods, with DNA quantification exhibiting the most robust overall performance and maintaining consistent accuracy across different CD4^+^ T cell count subgroups. The simulated multiple-testing strategy revealed that the ELISA-DNA tandem protocol achieved an optimal balance between sensitivity and testing efficiency. Notably, DNA and RNA quantification methods successfully resolved WB-indeterminate samples, highlighting their clinical utility in complex diagnostic scenarios.

The study identified one false-negative result in both RNA quantification and qualitative methods from a patient who later confirmed 1 month of ART treatment prior to sampling, despite self-reported non-use of ARVs. This observation aligns with the viral suppression effect of effective ART, where viral load rapidly declines below detection limits [21]. In contrast, DNA quantification remained positive in this case, confirming the stability of HIV proviral DNA integrated into the host genome [22]. Beyond ARV interference, the clinical reliability of RNA assays is inherently constrained by pre-analytical vulnerabilities, as viral RNA is highly susceptible to degradation if plasma separation or cold-chain transport is delayed in routine practice. Conversely, integrated proviral DNA offers significantly greater pre-analytical stability, further reinforcing its robustness as a confirmatory tool. Intriguingly, the single ELISA false-negative case occurred in an individual with high viral load and low CD4^+^ T cell counts. This paradoxical result may be attributed to the Hook effect, where excessive antigen-antibody complexes obscure binding sites [23], compounded by potential B-cell dysfunction and impaired humoral immunity in immunocompromised states [24]. Regarding specificity, RNA and DNA quantification showed no false positives, consistent with the high specificity of nucleic acid testing reported in previous studies [25–27]. ELISA’s false positives may relate to endogenous interferents or vaccination-induced cross-reactivity [28]. Conversely, the higher rate of false positives observed with the RNA qualitative assay can be largely attributed to its original kit design, with the manufacturer’s instructions confirming that this assay was designed for large-scale blood bank pool screening, prioritizing analytical sensitivity to prevent missed infections, thereby inherently compromising clinical specificity when repurposed for individual diagnosis. These findings underscore the necessity to account for ARV treatment history and carefully select assays tailored to clinical confirmation.

Simulated diagnostic strategies showed high net sensitivity and specificity across all tandem protocols, aligning with the improvements in diagnostic accuracy enabled by advances in NAT [27]. The ELISA-DNA combination achieved maximal net sensitivity (100% specificity), with only one positive sample failing dual positivity confirmation. This advantage stems from DNA testing’s high specificity for integrated proviral DNA [29], particularly effective in distinguishing true infections from ELISA false positives. While fifth-generation ELISA has reduced the diagnostic window to less than 2 weeks through separate antibody/p24 antigen detection [30], its sensitivity remains inferior to NAT in early acute infection. NAT-based initial screening strategies (DNA-RNA quantification or DNA-RNA qualitative combinations) achieved optimal performance with minimal testing iterations, circumventing antibody response variability [20]. Recent advances in point-of-care testing have further enhanced NAT accessibility [31], providing sustained technological support for NAT applications in HIV diagnosis. Crucially, while the practical implementation of DNA quantification is often presumed to be constrained by greater complexity and resource limitations, the actual patient-end cost of HIV DNA quantification at our institution is comparable to that of RNA quantitative testing. Therefore, integrating DNA quantification as a targeted confirmatory step following ELISA screening may not significantly increase the economic burden while offering a practical, highly robust alternative, particularly for vulnerable populations or individuals with undisclosed ARV exposure. In neonates born to HIV-positive mothers, maternal antibodies can persist for up to 18 months, rendering traditional serological tests inconclusive, thereby establishing NAT as the gold standard for early infant diagnosis [32]. Similarly, pregnant women require rapid and definitive confirmation to timely initiate prevention of mother-to-child transmission protocols. In these scenarios, the stability and accuracy of DNA testing are uniquely valuable.

Analysis of WB-indeterminate samples further revealed NAT’s diagnostic superiority. WB indeterminacy typically arises from incomplete antibody responses in early infection or nonspecific reactions [20], whereas ELISA may yield false positives due to autoimmune diseases, pregnancy, malignancies, or cross-reactivity with other viruses [28]. NAT directly targets viral nucleic acids detectable within 3–5 days post-infection [20], circumventing the antibody window period and remaining unaffected by host immune status. Notably, HIV DNA maintains stable detectability even when RNA viral load falls below detection limits due to PrEP/PEP or standardized ART regimens, as DNA integrates into the host genome [29]. This study further confirmed DNA testing’s stability following ART-mediated viral suppression, positioning it as a powerful alternative to RNA quantification for patients with prior ART exposure. Beyond the NAT techniques employed in this research, emerging 2024 technologies like CRISPR-Cas12a coupled with nested PCR have enhanced NAT sensitivity (95%) and specificity (100%) [33]. Our findings validate NAT’s pivotal role in resolving WB indeterminacy by shortening the window period, improving acute infection detection sensitivity, and reducing misinterpretation risks through standardized protocols. With increasing PCR accessibility and cost reduction, NAT may potentially supplant WB as the diagnostic cornerstone, enabling earlier case identification, accelerated diagnosis, and essential technical support for optimizing global HIV diagnostic pathways toward achieving the goal of ending AIDS epidemics.

This study presents a multi-method evaluation to comprehensively assess HIV detection methodologies. The robust performance of DNA quantification not only addresses the window-period limitations of conventional antibody testing but also provides a reliable solution for indeterminate WB interpretations. Furthermore, the ELISA-DNA tandem protocol offers evidence-based guidance for optimizing diagnostic workflows. However, limitations should be acknowledged: First, the single-center cohort with male predominance may compromise the stability of subgroup analyses, necessitating multicenter studies with larger cohorts to validate the generalizability of findings across broader demographic diversity, including vulnerable populations such as pregnant women and newborns. Second, the exclusion of acute-phase infections and antiviral therapy recipients restricts the evaluation of diagnostic efficacy under viral suppression or proviral DNA integration conditions, potentially overestimating method performance in real-world clinical complexity. Third, the unaddressed parameters of detection timeliness, operational complexity, and cost-effectiveness analysis may hinder the adoption of novel methodologies like DNA quantification in primary care settings. This work represents an advance in biomedical science because it validates DNA quantitative testing as a highly accurate HIV confirmatory tool that resolves diagnostic ambiguities.

## Conclusion

This study systematically evaluated the performance of four detection methods in HIV initial diagnosis, demonstrating that DNA quantitative detection exhibits robust sensitivity and specificity. It shows substantial diagnostic value in WB-indeterminate samples regardless of CD4^+^ T cell levels, providing critical evidence for optimizing current diagnostic protocols. Notably, the ELISA-DNA tandem strategy achieved 100% specificity while maintaining high sensitivity. While operational complexity may restrict its universal application in resource-limited primary care settings, the low testing frequency and high diagnostic efficiency balance accuracy with resource consumption, making it particularly suitable for supplemental confirmation following HIV screening in China. Notably, DNA testing successfully identified one additional ARV-treated case with high CD4 levels compared to RNA detection, resolving diagnostic challenges for RNA-suppressed infections where ARV usage history is undisclosed. These findings not only offer actionable pathways to achieve the diagnostic rate improvement goals outlined in China’s AIDS Prevention and Control Plan (2024-2030) but also validate the scientific rationale for implementing NAT as a targeted confirmatory method to accurately resolve WB-indeterminate misclassifications. Although higher costs and technical requirements may limit universal DNA testing adoption as a routine screening algorithm, its targeted application as a supplemental tool for WB-indeterminate cases and individuals with potential ART exposure holds significant clinical value, effectively shortening diagnostic windows, reducing late detection rates, and contributing substantially to curbing HIV transmission chains and alleviating disease burden.

## Summary table

### What is known about this subject

- Late HIV diagnosis remains a global challenge due to the limitations in current diagnostic testing strategies
- Western blot confirmation often yields indeterminate results, which significantly delays definitive diagnosis.
- While nucleic acid testing is accurate, direct evidence for its routine use in initial HIV confirmation is limited.

### What this paper adds

- DNA quantitative testing achieves 100% sensitivity and specificity in initial clinical HIV diagnosis.
- DNA testing successfully resolves Western blot indeterminacy and overcomes antiviral treatment interference.
- An ELISA-DNA tandem strategy optimizes testing efficiency and diagnostic accuracy to reduce late diagnosis.

## Data Availability

The original contributions presented in the study are included in the article/Supplementary Material, further inquiries can be directed to the corresponding authors.
