# Supplementary material for: Nucleic acid testing-optimized HIV diagnostic algorithms: a multimethod comparative effectiveness analysis in clinical practice
Source: Br J Biomed Sci. 2026 Jul 29;83:15887. doi: 10.3389/bjbs.2026.15887 (PMC13461564; doi:10.3389/bjbs.2026.15887)
Supplement: Supplementary file 1 [file Supplementaryfile1.docx]

**Supplementary Materials 1**

**Content**

[**Explanation of Data Availability	2**](#_Toc235544858)

[**Table S1: Cochran's Q and McNemar Test for Sensitivity Differences Among Four HIV Assays (n=102 Positive Samples)	3**](#_Toc235544859)

[**Table S2 Paired-sample area difference under the ROC curves	4**](#_Toc235544860)

[**Table S3 Inter-method and gold standard agreement analysis using Kappa for HIV RNA quantification, qualitative RNA detection, DNA quantification, and ELISA in first-time diagnostic testers	5**](#_Toc235544861)

[**Table S4 Positive detection counts among four assays in 102 HIV-positive specimens stratified by CD4⁺ T cell count	6**](#_Toc235544862)

[**Table S5 Results of Cochran's Q Test by CD4^+^ T cell	6**](#_Toc235544863)

[**Table S6 The performance of different testing combinations	7**](#_Toc235544864)

# Explanation of Data Availability

**Demographic information, Western Blot and CD4 Data**

Demographic information, Western blot results, and baseline CD4⁺ T-cell counts utilized in this analysis were retrieved directly from the hospital’s Electronic Medical Record system. Because this portion of the study involved a retrospective extraction from a de-identified clinical database rather than prospectively paired sampling, these specific clinical assessments were not performed independently by the research team. Consequently, baseline CD4⁺ T-cell counts were unavailable for 10 patients. This absence of data occurred because these individuals did not undergo synchronous immunological testing alongside their initial diagnostic workup due to inherent variations and scheduling constraints in routine clinical workflows.

Similarly, because EMR data acquisition is retrospective, raw experimental images, such as the original WB strips or primary instrument files, are not available for independent review. However, to ensure diagnostic rigor, all clinically recorded WB results were interpreted by the hospital's clinical laboratory, strictly adhering to the official National Guidelines for Detection of HIV/AIDS issued by the Chinese Center for Disease Control and Prevention. According to these national standards, an indeterminate result is defined as the presence of any HIV-1-specific band that do not meet the full criteria for a positive diagnosis.

# Table S1: Cochran's Q and McNemar Test for Sensitivity Differences Among Four HIV Assays (n=102 Positive Samples)

| **Comparison Groups** | **Inconsistency Test Results (n)** | **P value** |
| --- | --- | --- |
| Four methods (Cochran's Q) | - | 1.000^a^ |
| RNA quantification vs. RNA detection | RNA quantification(+)RNA detection(-): 0; RNA quantification(-)RNA detection(+): 0 | 1.000^b^ |
| RNA quantification vs. DNA | RNA quantification(+)DNA(-): 0; RNA quantification(-)DNA(+): 1 | 1.000^b^ |
| RNA quantification vs. ELISA | RNA quantification(+)ELISA(-): 1; RNA quantification(-)ELISA(+): 1 | 1.000^b^ |
| RNA detection vs. DNA | RNA detection(+)DNA(-): 0; RNA detection(-)DNA(+): 1 | 1.000^b^ |
| RNA detection vs. ELISA | RNA detection(+)ELISA(-): 1; RNA detection(-)ELISA(+): 1 | 1.000^b^ |
| DNA vs. ELISA | DNA(+)ELISA(-): 1; DNA(-)ELISA(+): 0 | 1.000^b^ |

a. Q = 1.286

b. McNemar Test

+: Positive; -: Negative

# Table S2 Paired-sample area difference under the ROC curves

| **Test Result Pair(s)** | **z** | **Sig. (2-tail)^a^** | **AUC Difference** | **Std. Error Difference^b^** | **95% CI** | |
| --- | --- | --- | --- | --- | --- | --- |
|  |  |  |  |  | **Lower Bound** | **Upper Bound** |
| RNA quantification - RNA detection | 6.074 | 0.000 | 0.124 | 0.161 | 0.084 | 0.164 |
| RNA quantification - DNA | -1 | 0.317 | -0.005 | 0.070 | -0.015 | 0.005 |
| RNA quantification - ELISA | 0.947 | 0.344 | 0.009 | 0.113 | -0.009 | 0.027 |
| RNA detection - DNA | -6.14 | 0.000 | -0.129 | 0.145 | -0.170 | -0.088 |
| RNA detection - ELISA | -5.031 | 0.000 | -0.115 | 0.170 | -0.160 | -0.070 |
| DNA - ELISA | 1.735 | 0.083 | 0.014 | 0.089 | -0.002 | 0.029 |

a. Null hypothesis: true area difference = 0

b. Under the nonparametric assumption

CI: Confidence Interval

# Table S3 Inter-method and gold standard agreement analysis using Kappa for HIV RNA quantification, qualitative RNA detection, DNA quantification, and ELISA in first-time diagnostic testers

| **ID** | **Comparison** | **Kappa** | **CI lower** | **CI upper** | **P value** | **Subjects** | **Strength** |
| --- | --- | --- | --- | --- | --- | --- | --- |
| 1 | All Four Methods | 0.850 | 0.789 | 0.890 | <0.001 | 215 | Almost Perfect |
| 2 | RNA quantification + RNA detection + DNA | 0.820 | 0.751 | 0.870 | <0.001 | 215 | Almost Perfect |
| 3 | RNA quantification + RNA detection + ELISA | 0.801 | 0.721 | 0.856 | <0.001 | 215 | Almost Perfect |
| 4 | RNA quantification + DNA + ELISA | 0.975 | 0.930 | 0.994 | <0.001 | 215 | Almost Perfect |
| 5 | RNA detection + DNA + ELISA | 0.801 | 0.720 | 0.856 | <0.001 | 215 | Almost Perfect |
| 6 | RNA quantification vs. Gold Standard | 0.991 | 0.972 | 1.000 | <0.001 | NA | Almost Perfect |
| 7 | RNA detection vs. Gold Standard | 0.733 | 0.648 | 0.815 | <0.001 | NA | Substantial |
| 8 | DNA vs. Gold Standard | 1.000 | 1.000 | 1.000 | <0.001 | NA | Almost Perfect |
| 9 | ELISA vs. Gold Standard | 0.972 | 0.934 | 1.000 | <0.001 | NA | Almost Perfect |
| 10 | RNA quantification vs. RNA detection | 0.743 | 0.663 | 0.828 | <0.001 | 215 | Substantial |
| 11 | RNA quantification vs. DNA | 0.991 | 0.972 | 1.000 | <0.001 | 215 | Almost Perfect |
| 12 | RNA quantification vs. ELISA | 0.963 | 0.924 | 0.991 | <0.001 | 215 | Almost Perfect |
| 13 | RNA detection vs. DNA | 0.733 | 0.648 | 0.815 | <0.001 | 215 | Substantial |
| 14 | RNA detection vs. ELISA | 0.705 | 0.604 | 0.796 | <0.001 | 215 | Substantial |
| 15 | DNA vs. ELISA | 0.972 | 0.934 | 1.000 | <0.001 | 215 | Almost Perfect |

# Table S4 Positive detection counts among four assays in 102 HIV-positive specimens stratified by CD4⁺ T cell count

| **CD4^+^ T cell (cells/µL)** | **True Positive** | **Number of Positive Test results** | | | |
| --- | --- | --- | --- | --- | --- |
|  |  | **RNA quantification** | **RNA detection** | **DNA** | **ELISA** |
| >350 | 42 | 41 | 41 | 42 | 42 |
| 200-350 | 31 | 31 | 31 | 31 | 31 |
| <200 | 19 | 19 | 19 | 19 | 18 |
| Missing | 10 | 10 | 10 | 10 | 10 |
| **Total** | 102 | 101 | 101 | 102 | 101 |

# Table S5 Results of Cochran's Q Test by CD4^+^ T cell

| **CD4^+^ T cell (cells/µL)** | **Q value** | **P value** | **Conclusion** |
| --- | --- | --- | --- |
| >350 | 3.000 | 1.000 | Not Significant |
| 200-350 | - | - | Fully Consistent |
| <200 | 3.000 | 1.000 | Not Significant |
| Missing | - | - | Fully Consistent |

# Table S6 The performance of different testing combinations

| **Combination** | **Testing Strategy** | **Net Sensitivity (%)** | **Net Specificity (%)** | **Tests Required** | **Number of Double-Positive Cases** |
| --- | --- | --- | --- | --- | --- |
| ELISA➡️RNA detection | Tandem | 98.05 | 99.56 | 215+103=318 | 100 |
| ELISA➡️RNA quantification | Tandem | 98.05 | 100.00 | 215+103=318 | 100 |
| ELISA➡️DNA quantification | Tandem | 99.02 | 100.00 | 215+103=318 | 101 |
| RNA detection➡️DNA quantification | Tandem | 99.02 | 100.00 | 215+129=344 | 101 |
| RNA quantification➡️DNA quantification | Tandem | 99.02 | 100.00 | 215+101=316 | 101 |
| RNA detection➡️RNA quantification | Tandem | 98.05 | 100.00 | 215+129=344 | 101 |
| RNA quantification➡️RNA detection | Tandem | 98.05 | 100.00 | 215+101=316 | 101 |
| DNA quantification➡️RNA detection | Tandem | 99.02 | 100.00 | 215+102=317 | 101 |
| DNA quantification➡️RNA quantification | Tandem | 99.02 | 100.00 | 215+102=317 | 101 |
